# ParAlleL: A Novel Population-Based Approach to Biological Logic Gates

**DOI:** 10.3389/fbioe.2019.00046

**Published:** 2019-03-21

**Authors:** Felipe A. Millacura, Brendan Largey, Christopher E. French

**Affiliations:** School of Biological Sciences, Institute of Quantitative Biology, Biochemistry and Biotechnology, University of Edinburgh, Edinburgh, United Kingdom

**Keywords:** *Escherichia coli*, 3-bits, full adder, full subtractor, calculator-like display, parallel approach

## Abstract

*In vivo* logic gates have proven difficult to combine into larger devices. Our cell-based logic system, ParAlleL, decomposes a large circuit into a collection of small subcircuits working in parallel, each subcircuit responding to a different combination of inputs. A final global output is then generated by a combination of the responses. Using ParAlleL, for the first time a completely functional 3-bit full adder and full subtractor were generated using *Escherichia coli* cells, as well as a calculator-style display that shows a numeric result, from 0 to 7, when the proper 3 bit binary inputs are introduced into the system. ParAlleL demonstrates the use of a parallel approach for the design of cell-based logic gates that facilitates the generation and analysis of complex processes, without the need for complex genetic engineering.

## Introduction

A major challenge in the field of synthetic biology is the construction of complex logic circuits that analyze variables as in electronics; where a single circuit accepts one or more binary inputs to generate one or more binary outputs. A cell-based logic network consists of engineered cells producing an output macromolecule only if the corresponding pattern of inputs is present. The mechanism of analysis is commonly based on the use of transcriptional regulators, transcription factors, polymerases, receptors, or recombinases (Brenner et al., 2018). Some examples of genetic circuits mimicking computational behavior are toggle switches, oscillators, boolean logic gates, feedback controllers, and multiplexers. Although there are genetic circuits that simulate computational behavior, the complex engineering of their biological chassis is affected by gene expression noise, mutation, cell death, undefined, and changing extracellular environments and improper interactions with the cellular context (Andrianantoandro et al., 2006). Furthermore, complex genetic engineering is necessary when multiple input variables are analyzed, limiting the processing capacity of the system.

Biological multiplexers analyze one or more signals over a common transmission line using interconnected transcription factors, recombinases, antisense RNA, or CRISPR-like technology (Nielsen and Voigt, 2014; Roquet et al., 2016; Brenner et al., 2018). However, complex genetic engineering is needed for wiring the basic computational units, becoming inefficient for moving beyond simple NOT or AND logic gates or for scaling to 3 bit logic circuits. The complexity of the genetic engineering required can be reduced by using distributed logic circuits, where the computation is distributed among several physically separated cellular consortia that each sense only one signal and respond by secreting a communication molecule (Regot et al., 2011). As a circuit responds to one signal, but not another, due to spatial distribution, a change in the state of the system can be triggered as response, making synthetic learning possible (Macia et al., 2017; Shipman et al., 2017). Even though the consortium approach makes Boolean circuit design simpler, it still shows a slow response and considerable complexity since each cell needs to recognize, synthesize and secrete a wiring molecule (Macia et al., 2016).

Here we propose an alternative logic architecture, which decomposes a large circuit into a collection of small subcircuits acting in parallel (hereafter ParAlleL). Rather than having a single type of agent (such as a genetically engineered cell) doing the computation, ParAlleL has separate types of agent that each react to a different combination of inputs. A final output is then generated by combination of the responses, making all kinds of binary operation possible. As an example, here we show the implementation of this concept using cells resistant to different combinations of antibiotics, with the response indicated by growth. This is used to demonstrate a completely functional 3 bit full adder and full subtractor, as well as a calculator-style display that shows digits from 0 to 7 based on three binary input bits.

## Methodology

### Reagents and Stock Solution Preparations

Antibiotic stock solutions were prepared as follows: 100 mg/ml carbenicilin disodium salt (Sigma-Aldrich #C1389), 50 mg/ml kanamycin sulfate (PanReac Applichem #A1493), 20 mg/ml chloramphenicol (Acros Organics #22792), 10 mg/ml tetracycline hydrochloryde (Duchefa Biochemie #T0150), 10 mg/ml gentamicin sulfate (Melford #G0124), and 50 mg/ml spectinomycin.HCl (LKT Labs #S6018). Developing solution contained 0.1 %w/v bromothymol blue (Sigma-Aldrich #114421) and 400 mM Trizma base pH7.5 (Sigma-Aldrich #T1503).

### Generation of Subcircuit Cells

*E. coli* JM109 was transformed with 200–300 pg of plasmid pSB4A5 (AmpR) or pSB4C5 (ChlR) (Registry of Standard Biological Parts) and selected on 100 μg/ml carbenicilin (Am) or 20 μg/ml chloramphenicol (Ch), respectively. Cells carrying the first bit plasmid were made chemically competent (Chung et al., 1989) and transformed with 200–300 pg of the 2nd bit plasmid, pSB1T3 (TetR) or pSB1K3 (KanR) (Registry of Standard Biological Parts). Selection was performed with the first antibiotic (Am or Ch) and the addition of 10 μg/ml Tetracycline (Tc) or 50 μg/ml kanamycin (Km), obtaining the two-bit combinations Km/Am (KA), Tc/Am (TA), Km/Ch (KC), and Tc/Ch (TC). This set of strains is sufficient to implement all two-bit binary operations.

The third bit layer was generated by transforming these four strains with pSEVA631 (GenR) (Silva-Rocha et al., 2012; GenBank JX560348) or pMO9075 (SpmR) (Keller et al., 2011). Resulting strains were selected on the 2-bit antibiotic combinations plus 10 μg/ml gentamicin (Gm) or 50 μg/ml spectinomycin (Sm). This gave 8 strains, designated ATG (Am/Tc/Gm), AKG (Am/Km/Gm), ATS (Am/Tc/Sm), AKS (Am/Km/Sm), CTG (Ch/Tc/Gm), CTS (Ch/Tc/Sm), CKG (Ch/Km/Gm), CKS (Ch/Km/Sm) based on their resistance markers. This set of strains is sufficient to implement all three-bit binary operations. Plasmid specifications are listed in Figure S2, Tables S1, S3, with further information about these antibiotics in Table S2. Plasmid sequences are available in different formats at https://doi.org/10.7488/ds/2497.

### Three-Bit Logic Operations

Tests were performed in 96-well microplates by inoculating cells (1:100) in LB broth (100 μL) supplemented with 1%w/v d(+)-glucose (Fisher Chemical #G0500). Plates were incubated for 18 h at 37°C without shaking and then developed by addition of the developing solution (0.1%w/v bromothymol blue in 400 mM Tris, pH7.5) in a ratio 1:20. Images were obtained using a Kodak ESPC315 Flatbed scanner. Design of the calculator-like display, full adder, and subtractor are shown in **Figures 2**, **3** and in Supplementary material (Figure S3). Raw figures were deposited at https://doi.org/10.7488/ds/2497.

## Results

In the distributed logic system of ParAlleL each input bit has two forms, ZERO and ONE, each of which is essential to certain output agents and inhibitory to others. Thus each agent reacts only to a certain combination of input bits, allowing generation of any arbitrary pattern of outputs for any pattern of inputs. In the implementation shown here, each input bit comes in two forms, each being an antibiotic lethal to sensitive strains. In this case, bit A is represented by ampicillin for zero, chloramphenicol for one, bit B by kanamycin for zero, tetracycline for one, and bit C by gentamicin for zero, spectinomycin for one. Thus, four strains are needed to implement any operation with two input bits, and eight strains for three input bits. In contrast to other cell-based logic schemes, only very minimal genetic engineering is required, essentially transformation with 3 different antibiotic resistance markers.

**Figure 1:**
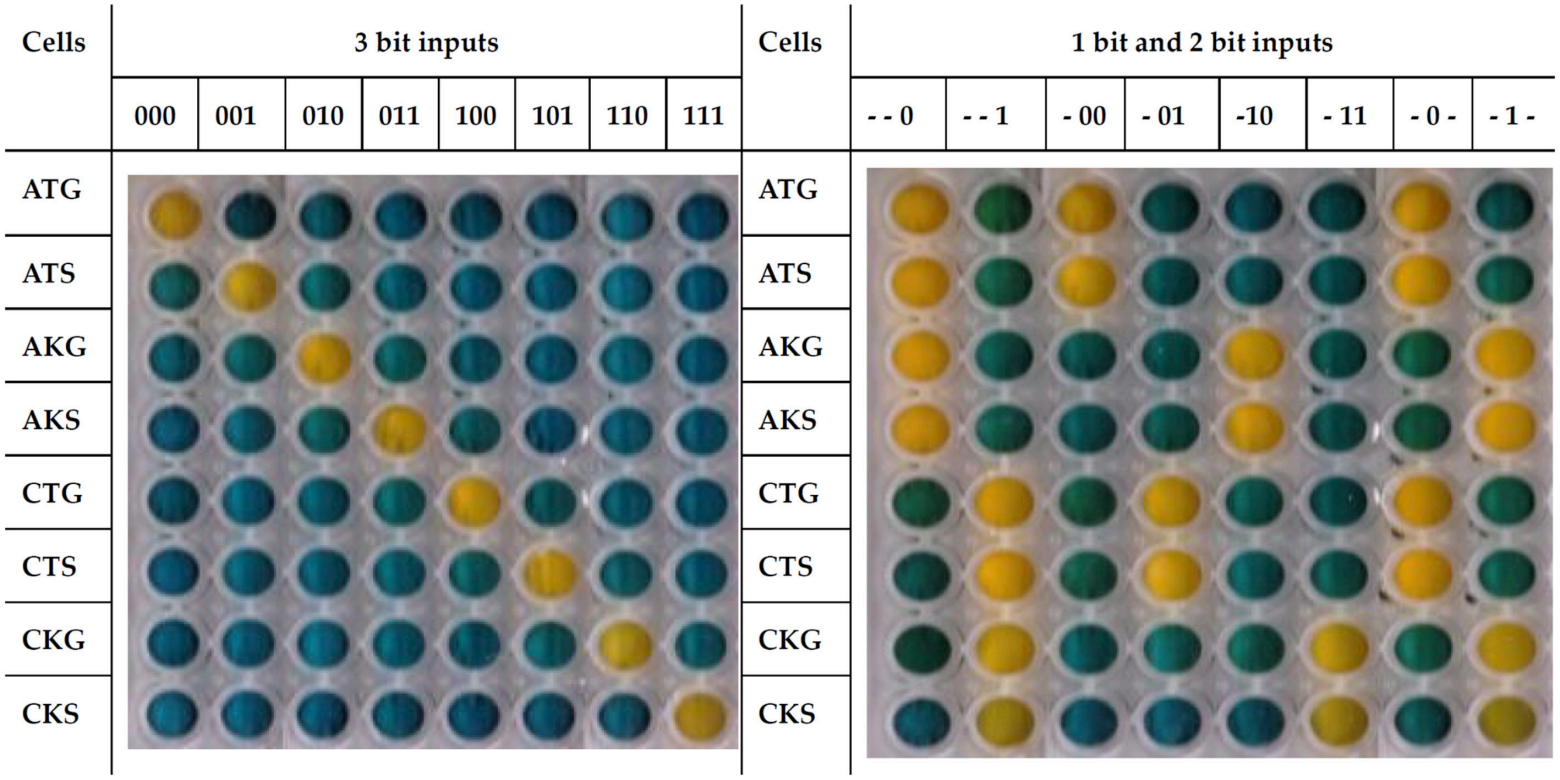
ParAlleL responding to 1 bit, 2 bit, and 3 bit inputs. ParAlleL subcircuit cells were spatially distributed in different wells (vertically) and exposed to specified 1 bit, 2 bit, or 3 bit inputs (top of each column). Cells were inoculated (1:100) in LB supplemented with 1% w/v glucose. After 18 h of incubation at 37°C, plates were developed by addition of 0.05 volumes of the developing solution.

Cells show a global response concordant with the behavior expected for a 1 bit, 2 bit, or 3 bit system (Figure 1). For instance, when the input 101 (chloramphenicol, tetracycline and spectinomycin) is added to the system growth is only observed in the corresponding CTS cells, which carry the proper resistance markers. The response time of the system is around 12 h (Figure S1) but plates were developed at 18 h to avoid false negatives or positives.

**Figure 2:**
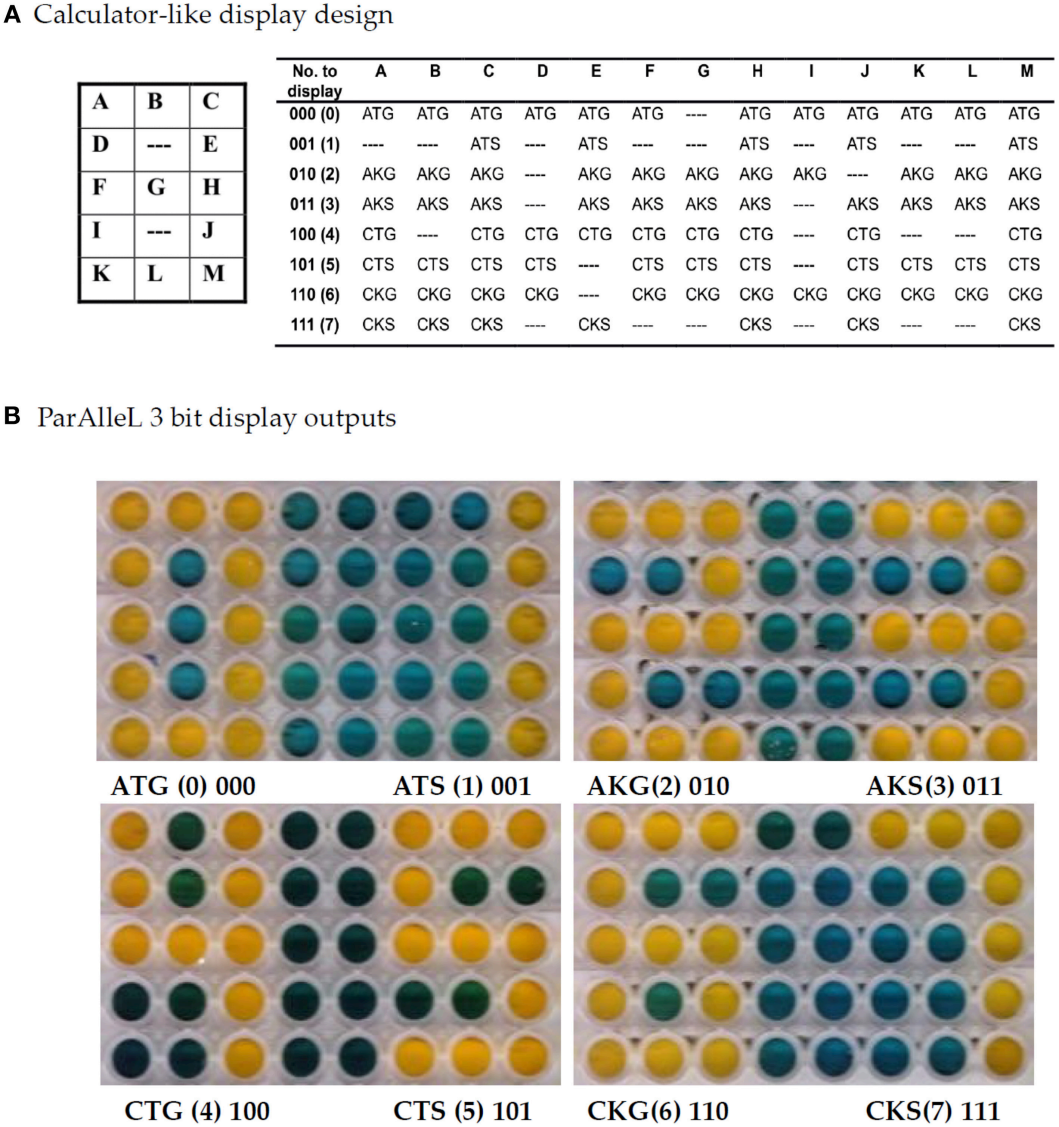
Digital calculator-like display using 3 bit ParAlleL. Figure shows all numerals from zero to seven based on the 8 binary inputs provided. **(A)** Subcircuit cells were mixed and distributed in a 3 × 5 matrix and inoculated (1:100) in LB supplemented with 1%w/v glucose. Plate was developed after 18 h of incubation, by addition of 0.05 volumes of developing solution. **(B)** Output number results obtained by addition of each 3 bit antibiotic combination.

In order to further test the ParAlleL system, a digital calculator-like display was designed (Figure 2A). In this case, multiple subcircuit cells are mixed in one well and the global response displays a number from 0 to 7 when the proper binary input is applied. Numbers represent the total eight possible values encoded within 3-bit binary inputs.

Input configuration versatility was proven by representing bit A in this case by gentamicin for zero, spectinomycin for one, bit B by kanamycin for zero, tetracycline for one, and bit C by ampicillin for zero, chloramphenicol for one. For instance, once input 110 represented by Ch/Km/Gm is added in the system, the number 6 is displayed (Figure 2).

**Figure 3:**
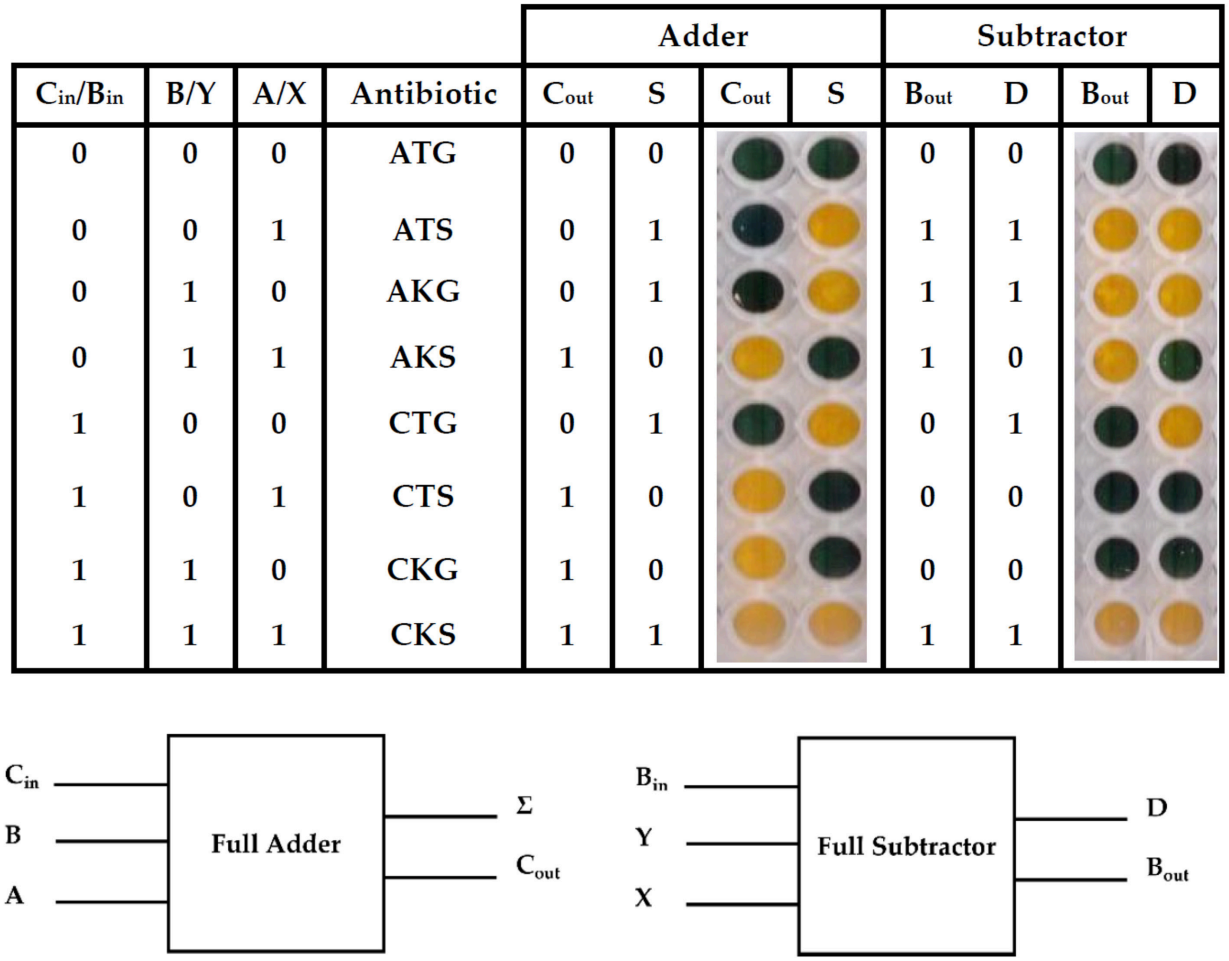
Full adder and subtractor using the 3 bit ParAlleL system. The figure shows results of addition and subtraction using the ParAlleL for 3 bit system. Cells were mixed as shown in Figure S3 and inoculated (1:100) in LB supplemented with 1%w/v glucose. After 18 hours of incubation, the plate was developed by addition of the 0.05 volumes of developing solution.

Finally, a full adder and a full subtractor were designed. A full-adder adds three binary inputs, often denoted as A, B, and C_in_, generating a Sum result (S) and a Carry-out (C_out_). A full subtractor, on the other hand, has a minuend (X), a subtrahend (Y) and an additional Borrow-in (B_in_) as inputs. The subtraction operation produces a difference (D) and a Borrow (B_out_) (Figure 3).

In order to generate the full adder and subtractor, multiple subcircuit cells were mixed and distributed in two different wells (Figure S3). One well represents the solution (S) or difference (D) and a second one the carry (C_out_) or borrow (B_out_), for the adder and subtractor, respectively (Figure 3). A yellow color represents growth and a positive output 1, a blue color represents no growth and a binary 0 output instead.

## Conclusions/Discussion

Subcircuits that solve complex calculations in parallel have been extensively used for computation in order to reduce the total computation time. Translating this approach to biological systems would allow us to analyze complex processes, currently difficult in synthetic biology, as multiple simple sub-circuits.

In our proof of concept, we present a biological information processing system, ParAlleL, capable of exploiting the parallelism in mixed bacterial cultures. ParAlleL decomposes the analysis of 2 and 3 bit complex inputs, into 4 and 8 sub circuits, respectively (Figure 1). Each sub-circuit corresponds to a different *E. coli* strain carrying a different combination of antibiotic resistance markers (Table S1). As an example, in the 3 bit system the input 000 is represented by the antibiotics ampicillin, tetracycline and gentamicin (Figure 1). When this input is entered into the system, all cells that are not encoded for responding to 000 will die, but cells carrying the proper plasmid combination, pSB4A5, pSB1T3, and pSEVA621 will not (Figures 1, 2), therefore, a live/dead response (output) is achieved in all sub circuits, the output of each well being one (growth) or zero (failure to grow) (Figure 2 and Figure S2).

ParAlleL uses cellular consortia instead of a single type of cell. A similar approach has been developed by Macia et al. (2016, 2017) using eukaryotic cells, and even showing the possibility of generating transient memory. However, that approach requires a sophisticated design as it relies on a secreted intermediate molecule (hormone-like) that must be kept at the right production level, and that should be previously activated by X (Repressor) and Y (SsrA-tagged protein) degradation. Furthermore, since the output of the circuit is distributed among different consortia, the concentration of the secreted molecule can differ according to the number of cells simultaneously producing it. This kind of multicellular approach and others based on single cells require sophisticated wiring design (Silva-Rocha and de Lorenzo, 2008; Siuti et al., 2013; Macia et al., 2016, 2017). By contrast, ParAlleL requires very minimal genetic modification and little tuning to obtain reliable outputs (Figures 2, 3).

The implementation of ParAlleL presented here is simple, but its further development to useful applications presents a number of challenges. Firstly, expansion to 4 bits and beyond would require further well-behaved and non-cross-reacting antibiotic resistance markers, and would probably lead to even greater disparities in growth rate than those observed in the three-bit system (Figure S1). This could be addressed, and the flexibility and usefulness of the system increased, by moving away from direct use of antibiotics to a system using tightly controlled inducible promoters, each controlling a lethal “death gene,” such as *ccdB*, with either the presence or absence of the inducer leading to lethality. In this way the system could be made to respond to different combinations of useful inputs, for example in the construction of multiplexed biosensors.

However, to achieve the full potential of ParAlleL, it will be necessary to generate layered systems in which the output from one layer serves as input to another layer. This might be accomplished via quorum sensing, but the implementation would be rather complex and limited. A more attractive option is to transfer the same concept, using a set of agents which each responds to a single combination of inputs, to an alternative system. For example, the concept could be implemented in a cell-free system, in which inputs may be present as small molecules interacting with transcription factors, or as DNA or RNA oligonucleotides. Outputs from the first layer, in the form of DNA or RNA molecules generated by DNA replication (e.g., PCR) or transcription, could then serve as inputs to a second layer. For example, in a PCR-based approach, one of a set of templates would be amplified based on which primer oligonucleotides were added; the output PCR product could then be processed to generate another set of oligonucleotides, or used directly to initiate priming on a second set of templates via formation of three-way junctions. Alternatively, in a transcription-based system, output RNAs from a first layer computation could act as guide RNAs directing binding of CRISPR-based transcription factors to further templates to generate a new set of guide RNAs, eventually leading to production of an output RNA such as a mRNA leading to translation of a visually detectable signal.

Another interesting aspect of ParAlleL is that, since the final output is the result of a population-based calculation, these systems may show a level of “Byzantine fault tolerance,” allowing reliable outcomes even in the face of levels of noise which are unavoidable in biological systems. This would represent a new level of robustness in biological computation systems.

## Data Availability

All datasets generated for this study are included in the manuscript and/or the supplementary files. More Data is available at https://doi.org/10.7488/ds/2497.

## Author Contributions

FM and CF conceived and designed the experiments. FM and BL performed the experiments. FM and CF analyzed and validated the data. FM and CF contributed reagents, materials, and analysis tools. FM and CF wrote—reviewed & edited the manuscript.

### Conflict of Interest Statement

The authors declare that the research was conducted in the absence of any commercial or financial relationships that could be construed as a potential conflict of interest.
