# Supplementary material for: ParAlleL: A Novel Population-Based Approach to Biological Logic Gates
Source: Front Bioeng Biotechnol. 2019 Mar 21;7:46. doi: 10.3389/fbioe.2019.00046 (PMC6437039; doi:10.3389/fbioe.2019.00046)
Supplement: Supplementary file 1 [file Data_Sheet_1.pdf]

Supplementary data

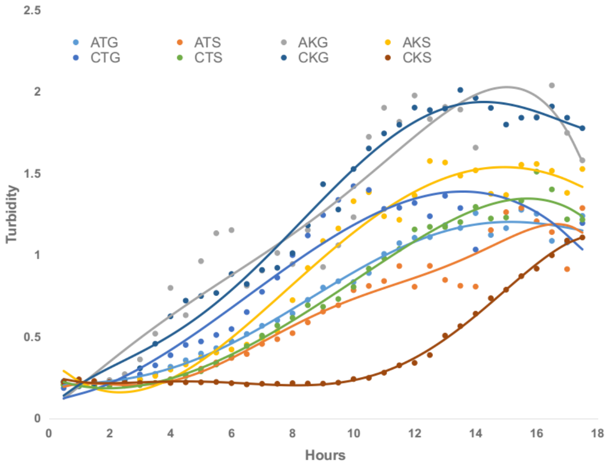

Figure S1. ParAlleL subcircuit cell growth curves. Growth curves of the 3-bit subcircuit cells in LB + 0.1% glucose with their respective antibiotic combinations. **A:** Carbenicilin (100 µg/mL), **C:** Chloramphenicol (20 µg/mL) **T:** Tetracycline (10 µg/mL), **K:** Kanamycin (50 µg/mL), **G:** Gentamicin (10 µg/mL). **S:** Spectinomycin (50 µg/mL). Overnight culture (0.01 volume) was used as inoculum.

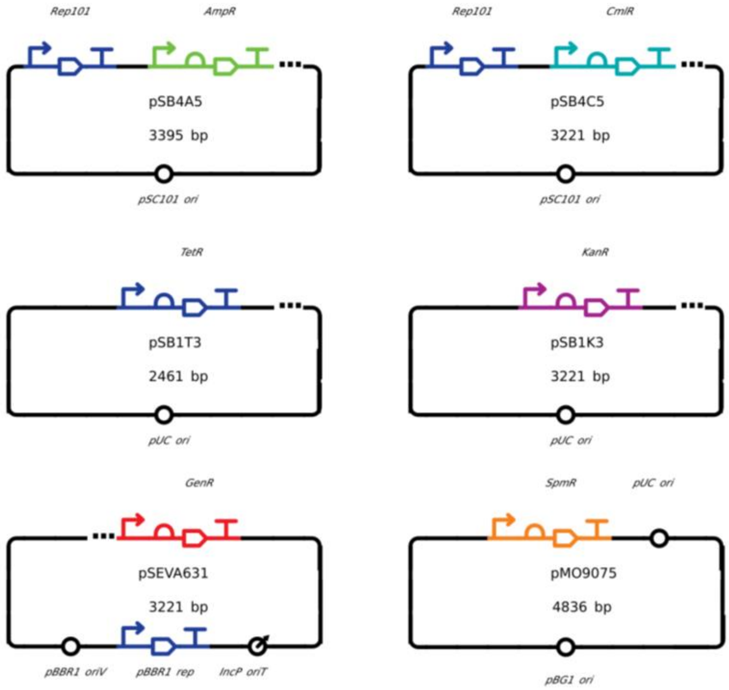

Figure S2. Plasmids carried by subcircuit cells. Plasmids carrying antibiotic resistances for Ampicillin (pSB4A5); Chloramphenicol (pSB4C5); Tetracycline (pSB1T3); Kanamycin (pSB1K3); Gentamicin (pSEVA631) and Spectinomycin (pMO9075) represented in SBOL format. Plasmid sequences available at https://doi.org/10.7488/ds/2497.

Figure S3: ParAlleL 3 bit Full adder/subtractor design

		Adder		Subtractor	
**Input**	**Antibiotic**	C_{out}	**S**	B_o	**D**
**000**	**ATG**	----	----	----	----
**001**	**ATS**	----	+++	+++	+++
**010**	**AKG**	----	+++	+++	+++
**011**	**AKS**	+++	----	+++	----
**100**	**CTG**	----	+++	----	+++
**101**	**CTS**	+++	----	----	----
**110**	**CKG**	+++	----	----	----
**111**	**CKS**	+++	+++	+++	+++

Cells added in specified wells carry resistance markers for:

ATG: Ampicilin, Tetracycline, Gentamicin.

ATS: Ampicilin, Tetracycline, Spectinomycin.

AKG: Ampicillin, Kanamycin, Gentamicin.

AKS: Ampicillin, Kanamycin, Spectinomycin.

CTG: Chloramphenicol, Tetracycline, Gentamicin.

CTS: Chloramphenicol, Tetracycline, Spectinomycin.

CKG: Chloramphenicol, Kanamycin, Gentamicin.

CKS: Chloramphenicol, Kanamycin, Spectinomycin.

Additional information

Table S1: Plasmids used for generating ParAlleL subcircuit strains.

Plasmid	Antibiotic marker	ORI	Copy number	Reference
pSB4A5	AmpR	pSC101	5	http://parts.igem.org/Part:pSB4A5
pSB4C5	CmlR	pSC101	5	http://parts.igem.org/Part:pSB4C5
pSB1T3	TetR	pMB1(der)	100-300	http://parts.igem.org/Part:pSB1T3
pSB1K3	KanR	pMB1 (der)	100-300	http://parts.igem.org/Part:pSB1K3
pSEVA631	GenR	pBBR1	medium	https://www.ncbi.nlm.nih.gov/nuccore/JX560348
pMO9075	SpmR	pBG1	low	Keller, et al., 2011

Table S2: Antibiotics and resistance cassettes used on ParAlleL.

Antiobiotic	Class	Mode of action	Resistance
Ampicillin	β-lactam	Bactericidal; Inhibits cell wall synthesis	β-lactamase (bla) gene
Kanamycin	Aminoglycoside	Bactericidal; Binds 30S ribosomal subunit; causes mistranslation	Neomycin phosphotransferase II
Chloramphenicol	Chloramphenicol	Bacteriostatic; Binds 50S ribosomal subunit; inhibits peptidyl translocation	Chloramphenicol acetyl transferase
Tetracycline	Tetracycline	Bacteriostatic; Binds 16S ribosomal subunit; inhibits protein synthesis (elongation step)	Tetracycline efflux protein
Gentamicin	Aminoglycoside	Irreversibly binding the 30S subunit of the bacterial ribosome	Gentamicin-3-N-acetyltransferase
Spectinomycin	Aminocyclitol	It binds to the 30S and interrupts protein synthesis affecting 16S rRNA	Spectinomycin adenyltransferase

Table S3: Plasmid incompatibility groups.

Incompatibility group	Regulation	Comment
pBR322/ColE1/pMB1	Inhibitor-target RNA I	Control processing of pre-RNAII into primer
IncFII, pT181	RNA	Affecting synthesis of RepA protein
R6K*, P1, F, pSC101, Rts1, P15A*, RK2	Iteron binding	Sequestering of RepA protein
